# Fixed Monthly versus Less Frequent Ranibizumab Dosing and Predictors of Visual Response in Exudative Age-Related Macular Degeneration

**DOI:** 10.1155/2012/690641

**Published:** 2012-11-28

**Authors:** Seenu M. Hariprasad, Lawrence S. Morse, Howard Shapiro, Pamela Wong, Lisa Tuomi

**Affiliations:** ^1^Section of Ophthalmology and Visual Science, Department of Surgery, University of Chicago, 5841 S. Maryland Avenue, Chicago, IL 60637, USA; ^2^Department of Ophthalmology and Vision Science, University of California Davis Health System Eye Center, 4860 Y Street, Suite 2400 Sacramento, CA 95817, USA; ^3^Genentech Inc., 1 DNA Way, South San Francisco, CA 94080, USA

## Abstract

*Purpose*. To examine temporal patterns of visual acuity (VA) response to pooled 0.3 mg/0.5 mg ranibizumab treatment in patients with age-related macular degeneration and identify potential baseline predictors of response. *Design*. Retrospective analysis. *Methods*. Results from 1824 ranibizumab-treated patients receiving fixed monthly, quarterly, or as-needed dosing after three monthly loading doses in four phase III/IIIb trials (ANCHOR, MARINA, PIER, and SAILOR) were analyzed. *Results*. At month 3, 14.9% to 29.4% of patients had gained ≥15 letters. Not all patients achieved peak gains at month 3; many continued to have VA increases throughout treatment. After three monthly loading doses, continued monthly dosing resulted in further gains, as there were more delayed 15-letter responders at month 12 (14.7–16.1%) than with less frequent dosing (5.0–6.0%). Monthly dosing also resulted in more patients maintaining VA gains at later time points. Early 15-letter responders had lower baseline mean VA than delayed 15-letter responders in ANCHOR and MARINA; no other differences in baseline characteristics were noted. *Conclusions*. Although some patients have rapid improvements in VA, others do not experience peak VA until later during treatment. Continued monthly dosing resulted in a greater percentage of patients gaining ≥15 letters than with switching to less frequent dosing regimens.

## 1. Introduction

During the pivotal phase III clinical trials of ranibizumab for the treatment of age-related macular degeneration (AMD), MARINA (**M**inimally Classic/Occult Trial of the **A**nti-VEGF Antibody **R**anibizumab **I**n the Treatment of **N**eovascular **A**ge-Related Macular Degeneration) and ANCHOR (**AN**ti-VEGF Antibody for the Treatment of Predominantly Classic **CHOR**oidal Neovascularization in Age-Related Macular Degeneration), patients were treated with ranibizumab using a fixed monthly dosing regimen that resulted in 35.7% and 40.3% of patients in ANCHOR and 24.8% and 33.8% of patients in MARINA experiencing ≥15 letter visual acuity (VA) gains at month 12 (ranibizumab 0.3 mg and 0.5 mg, resp.) [1, 2]. At month 12, mean VA improvements were 8.5 and 11.3 letters from baseline in ANCHOR and 6.5 and 7.2 letters in MARINA (in patients treated with ranibizumab 0.3 mg and 0.5 mg, resp.) [1, 2]. Lack of clinically significant differences between the lower and higher doses of ranibizumab and rapid mean VA increases in the initial 3 months that were sustained in later months suggested that monthly dosing may have reached a “ceiling effect” with no additional improvement in VA possible at those doses. As a result, subsequent clinical trials explored the possibility that similar gains in VA could be achieved with less frequent dosing of ranibizumab 0.3 mg and 0.5 mg. In PIER (A **P**hase IIIb, Multicenter, Randomized, Double-Masked, Sham **I**njection-Controlled Study of the **E**fficacy and Safety of **R**anibizumab in Subjects with Subfoveal Choroidal Neovascularization [CNV] with or without Classic CNV Secondary to Age-Related Macular Degeneration), patients received monthly ranibizumab for three doses followed by quarterly dosing. This regimen resulted in a reduced treatment effect during quarterly dosing with mean decreases of 1.6 and 0.2 letters from baseline at month 12 (ranibizumab 0.3 mg and 0.5 mg, resp.), indicating that this reduced dosing frequency was less effective overall [3]. SAILOR (**S**afety **A**ssessment of **I**ntravitreous **L**ucentis f**OR** AMD) also employed an initial loading dose of three monthly ranibizumab injections, but with subsequent as-needed retreatment based on VA and/or optical coherence tomography (OCT) criteria [4]. Although some of the loss of treatment effect seen in PIER was prevented by as-needed dosing in SAILOR, mean improvement in VA was modest (0.5 and 2.3 letters gained from baseline at month 12; ranibizumab 0.3 mg and 0.5 mg, resp.) compared with ANCHOR and MARINA. Current practices tend to employ dosing strategies that differ from the recommended monthly treatment and are similar to those used in the PrONTO (**Pr**ospective **O**ptical Coherence Tomography Imaging of Patients with **N**eovascular AMD **T**reated with Intra**O**cular Ranibizumab) study (guided by VA and OCT measures with monthly monitoring) [5, 6] or treat-and-extend dosing (OCT guided, but with increasingly longer between-visit intervals if the patient has no signs of disease activity) [7] to maximize VA while reducing the number of injections and patient visits. 

 The purpose of the current analysis was to compare differences in the temporal patterns of VA response of pooled ranibizumab 0.3 mg/0.5 mg treatment in two clinical trials that employed fixed monthly dosing through the entire treatment period (ANCHOR and MARINA) with two clinical trials that employed less frequent dosing after three initial loading doses (PIER and SAILOR). Since not all patients achieve their peak VA at the same time point relative to the initiation of ranibizumab treatment, the temporal analysis was conducted to clarify when patients achieve their peak VA with fixed monthly dosing compared with less than monthly dosing. Another purpose of the current analysis was to identify potential baseline predictors of response to pooled ranibizumab 0.3 mg/0.5 mg treatment by comparing the baseline characteristics of patients who gained ≥15 letters from baseline as measured at month 3 (early 15-letter responders) and patients who did not gain ≥15 letters from baseline at month 3 but gained ≥15 letters from baseline as measured at month 12 (delayed 15-letter responders). 

## 2. Methods

### 2.1. Study Design

This retrospective analysis was conducted using data from four multicenter, randomized, phase III/IIIb clinical trials of intravitreal ranibizumab in patients with neovascular AMD. Detailed eligibility requirements for patients and eyes, clinical evaluation procedures, and clinical data collection methods and schedules for ANCHOR, MARINA, PIER, and SAILOR have been published [1–4]. Patients were treated with ranibizumab 0.3 mg or 0.5 mg with a fixed monthly dosing regimen (ANCHOR and MARINA) or with an initial three months of loading doses at months 0, 1, and 2 followed by quarterly dosing (PIER) or as-needed dosing based on VA and/or OCT findings (SAILOR cohort 1 treatment-naïve patients [Table 1]). For SAILOR, only cohort 1 treatment-naïve patients were included in this analysis to ensure that the patient population was most similar to the ANCHOR, MARINA, and PIER trials and that all trials began with three monthly loading doses as part of their dosing schedules.

Best-corrected VA in the study eye was assessed using the early treatment diabetic retinopathy study (ETDRS) chart at a distance of two meters (ANCHOR and MARINA) or four meters (PIER and SAILOR cohort 1). Baseline characteristics were compared between early 15-letter responders and delayed 15-letter responders to identify potential baseline predictors of response. Early 15-letter responders were defined as patients who gained ≥15 letters from baseline as measured at month 3 and delayed 15-letter responders were defined as patients who did not gain ≥15 letters from baseline at month 3 but gained ≥15 letters from baseline as measured at month 12. Month 3 was chosen for the first time point in this comparison because all ranibizumab-treated patients in these four clinical trials received three monthly loading doses at months 0, 1, and 2. After month 3, patients enrolled in PIER or SAILOR received ranibizumab at less frequent intervals (quarterly or criteria-based as needed, resp.). Month 12 was chosen as the second time point in this comparison because we were interested in the long-term response, and month 12 was the longest time point used across all four trials.

 For this analysis, VA outcomes were summarized for the pooled ranibizumab 0.3 mg/0.5 mg groups by each trial. Time to first gain of ≥15 letters from baseline was analyzed using Kaplan-Meier methods for the pooled ranibizumab group by trial. A comparison of baseline characteristics between early 15-letter responders and delayed 15-letter responders was conducted among pooled ranibizumab patients by trial. Means were compared using Student's *t*-tests or Satterthwaite's approximation *t*-test when the variances of the two groups were unequal. Percentages were compared using the Pearson chi-square test or Fisher's exact test when appropriate. Missing data were imputed using the last observation carried forward method. All analyses were conducted using SAS version 9.1 (SAS Institute Inc., Cary, NC, USA). No adjustments were made for multiple comparisons.

## 3. Results

**Figure 1:**
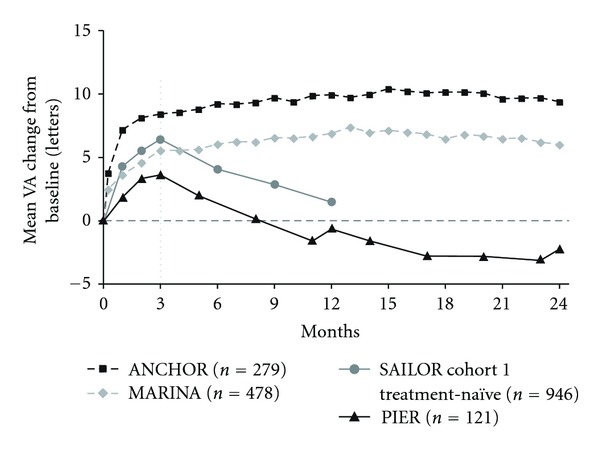
Overall mean change in visual acuity (VA) over time among pooled ranibizumab-treated patients by clinical trial. Dosing: ANCHOR and MARINA, monthly; PIER, months 0, 1, 2, 5, 8, 11, 14, 17, 20, and 23; SAILOR, months 0, 1, and 2, then as needed. Vertical line indicates switch from monthly to quarterly and as-needed dosing in PIER and SAILOR, respectively.

A total of 1824 ranibizumab-treated patients from the four clinical trials were included in this analysis; 757 patients received ranibizumab with fixed monthly dosing throughout their respective trial (ANCHOR or MARINA), while 1067 patients received three monthly loading doses of ranibizumab followed by quarterly or criteria-based as-needed dosing (PIER or SAILOR). The majority of the mean VA increase occurred during the first 3 months of each trial with monthly dosing (Figure 1). After month 3, this increase in mean VA was maintained when fixed monthly dosing was continued (ANCHOR and MARINA). In contrast, peak mean VA gain was achieved at month 3 (the last monthly injection) during PIER and SAILOR and thereafter decreased with no further increase after switching to quarterly or criteria-based as-needed dosing.

**Figure 2:**
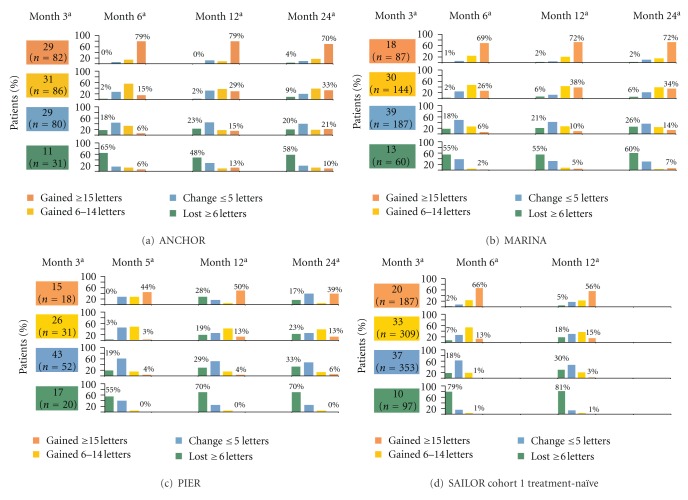
Distribution of visual acuity change from baseline based on visual acuity status at month 3 among pooled ranibizumab-treated patients by clinical trial. ^a^All visual acuity changes are compared with baseline. For ease of interpretation, all percentages are rounded to the nearest whole number.

The distributions of patients with ≥15 letter gain, 6 to 14 letter gain, ≤5 letter change (a change between  −5 and +5 letters), and ≥6 letters lost from baseline are shown in Figure 2 by month 3 VA classification. At month 3, 14.9% to 29.4% of patients had a ≥15 letter gain from baseline. Continuation of fixed monthly dosing after month 3 resulted in a greater percentage of early 15-letter responders maintaining their VA gain at later months than with quarterly dosing in PIER or criteria-based as-needed dosing in SAILOR. Similarly, greater percentages of patients who gained 6 to 14 letters at month 3 maintained those gains or had further gains in VA at month 6, 12, or 24 with fixed monthly dosing than with quarterly or criteria-based as-needed dosing. The percentage of patients who lost 6 or more letters at month 12 was smaller in the fixed monthly dosing studies (12.5% in ANCHOR and 17.4% in MARINA) compared with quarterly dosing (33.1% in PIER) and criteria-based as-needed dosing (26.3% in SAILOR).

**Figure 3:**
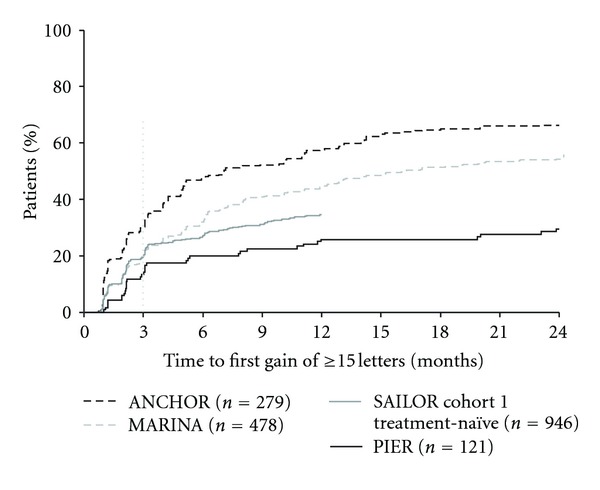
Time to first gain of ≥15 letters from baseline among pooled ranibizumab-treated patients by clinical trial. Dosing: ANCHOR and MARINA, monthly; PIER, months 0, 1, 2, 5, 8, 11, 14, 17, 20, and 23; SAILOR, months 0, 1, and 2, then as needed. Vertical line indicates switch from monthly to quarterly and as-needed dosing in PIER and SAILOR, respectively.

 Across the four clinical trials analyzed, some patients did not achieve their first gain of ≥15 letters from baseline until after month 3 (Figure 3). A greater percentage of patients who continued fixed monthly dosing after month 3 were delayed 15-letter responders (14.7% in ANCHOR and 16.1% in MARINA) than with quarterly (5.0%) or criteria-based as-needed (6.0%) dosing in PIER or SAILOR, respectively. Among patients who had an initial loss in VA of ≥6 letters at month 3, 5% to 13% of patients treated with continued, fixed monthly dosing gained ≥15 letters at month 12 or month 24, whereas no patients receiving quarterly and very few patients (~1%) receiving criteria-based as-needed dosing who had initially lost ≥6 letters at month 3 had ≥15 letter gains later during treatment at month 12 (Figure 2). 

**Figure 4:**
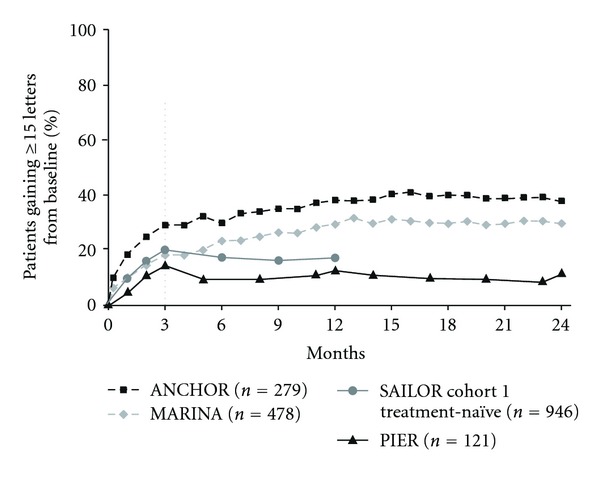
Percentage of patients gaining ≥15 letters from baseline over time among pooled ranibizumab-treated patients by clinical trial. Dosing: ANCHOR and MARINA, monthly; PIER, months 0, 1, 2, 5, 8, 11, 14, 17, 20, and 23; SAILOR, months 0, 1, and 2, then as needed. Vertical line indicates switch from monthly to quarterly and as-needed dosing in PIER and SAILOR, respectively.

**Figure 5:**
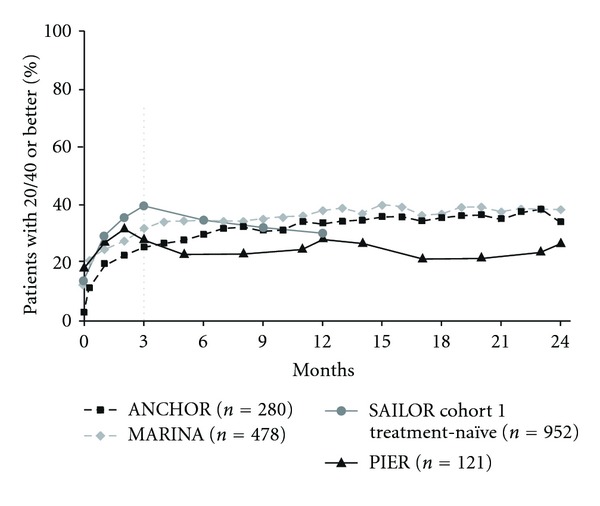
Percentage of patients with 20/40 or better vision over time among pooled ranibizumab-treated patients by clinical trial. Dosing: ANCHOR and MARINA, monthly; PIER, months 0, 1, 2, 5, 8, 11, 14, 17, 20, and 23; SAILOR, months 0, 1, and 2, then as needed. Vertical line indicates switch from monthly to quarterly and as-needed dosing in PIER and SAILOR, respectively. ANCHOR: *n* = 279 at month 0; SAILOR cohort 1 treatment-naïve: *n* = 946 at month 0.

**Figure 6:**
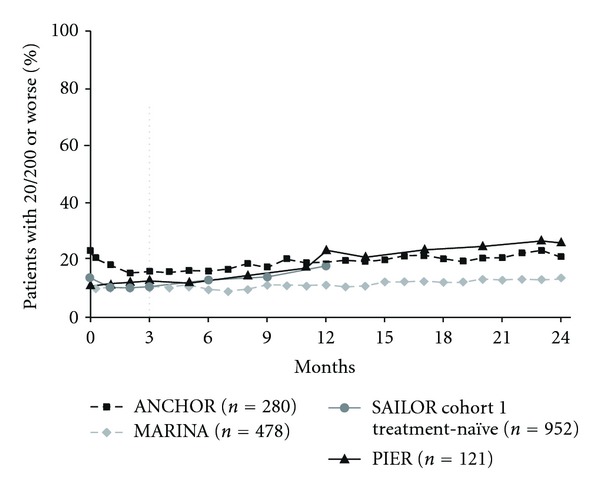
Percentage of patients with 20/200 or worse vision over time among pooled ranibizumab-treated patients by clinical trial. Dosing: ANCHOR and MARINA, monthly; PIER, months 0, 1, 2, 5, 8, 11, 14, 17, 20, and 23; SAILOR, months 0, 1, and 2, then as needed. Vertical line indicates switch from monthly to quarterly and as-needed dosing in PIER and SAILOR, respectively. ANCHOR: *n* = 279 at month 0; SAILOR cohort 1 treatment-naïve: *n* = 946 at month 0.

Other visual-outcome measures also showed similar patterns when responses to fixed monthly dosing and quarterly or criteria-based as-needed dosing were compared. The percentage of patients with ≥15 letter gain over time increased through month 3 (Figure 4) and continued to increase after month 3 when patients were maintained on fixed monthly dosing (ANCHOR or MARINA), but decreased with less frequent dosing (PIER or SAILOR). Similarly, after month 3, the percentage of patients with 20/40 or better vision increased with continued monthly dosing but not with less frequent dosing at month 12 (Figure 5). After month 3, the percentage of patients with 20/200 or worse vision was stable in ANCHOR and MARINA, but an increase was seen when patients received ranibizumab quarterly in PIER or criteria-based as-needed in SAILOR (Figure 6). 

 An analysis of the baseline characteristics from 374 early 15-letter responders and 181 delayed 15-letter responders was conducted to identify potential baseline predictors of response. Early 15-letter responders had lower baseline mean VA than delayed 15-letter responders in ANCHOR and MARINA (*P* < 0.05; Table 2). No other statistically significant differences in baseline characteristics were noted between early and delayed 15-letter responders. 

## 4. Discussion

In this retrospective analysis of data from four phase III/IIIb randomized, multicenter clinical trials of ranibizumab for the treatment of neovascular AMD, comparison of VA responses to different ranibizumab dosing regimens suggests that fixed monthly dosing provides greater overall improvement in VA than less than monthly dosing, on average. Mean VA increased significantly from baseline with three monthly loading doses in these four clinical trials. At month 12, the mean VA increases from baseline were maintained with continued fixed monthly dosing (ANCHOR: +8.5 and +11.3 letters; MARINA: +6.5 and +7.2 letters; ranibizumab 0.3 mg and 0.5 mg, resp.) compared with less frequent dosing (PIER:  −1.6 and  −0.2 letters; SAILOR: +0.5 and +2.3 letters; ranibizumab 0.3 mg and 0.5 mg, resp.) [1–4]. When analyzing the time to first gain of ≥15 letters, some patients experienced their first gain well after month 3, with a few patients achieving ≥15 letter gains within a few months of trial completion (month 12 in SAILOR and month 24 in ANCHOR, MARINA, and PIER). Thus, these results suggest that not all patients achieved optimal gains by month 3. After month 3, further improvement may be seen with continued fixed monthly dosing, but improvement may not be as pronounced if the patient is switched to less frequent dosing. Fixed monthly dosing resulted in proportionally more delayed 15-letter responders than with less frequent dosing. 

In the comparison of age-related macular degeneration treatments trials [CATT], a fixed monthly dosing of ranibizumab 0.5 mg was compared with the administration of ranibizumab 0.5 mg on an as-needed basis [8]. Patients in the as-needed group underwent time-domain OCT and were evaluated for treatment every 28 days. Treatment was administered if signs of active neovascularization (defined as fluid on OCT), new or persistent hemorrhage, decreased VA as compared with the previous examination, or dye leakage or increased lesion size on fluorescein angiography were present. Mean change (±standard error) in VA score from baseline was a gain of 8.5 ± 0.8 letters at week 52 in patients administered ranibizumab monthly, whereas patients administered ranibizumab on an as-needed basis had a mean gain of 6.8 ± 0.8 letters. Treatment outcomes were considered similar by the authors, because the 99.2% confidence interval for the mean change in VA score was between  −5 to  +5 letters, the prespecified noninferiority margin [8].

 Alternative as-needed dosing regimens have recently been investigated [9, 10], and a retrospective study by Gupta et al. has evaluated visual outcomes following a treat and extend approach [11], however, an optimal nonmonthly dosing regimen is yet to be defined. 

 Fixed monthly ranibizumab dosing may be important for the maintenance of improvement in VA. As noted, much higher percentages of patients with a ≥15 letter gain at month 3 who continued on fixed monthly dosing in ANCHOR or MARINA maintained VA improvement at months 12 and 24 compared with patients receiving quarterly or criteria-based as-needed dosing. In addition, fewer early 15-letter responders who continued with fixed monthly dosing had lost ≥6 letters at later time points than patients receiving less frequent dosing. Similarly, more patients with modest VA gains of 6 to 14 letters from baseline at month 3 ended their respective trials with this VA gain maintained or improved when continuing fixed monthly dosing compared with patients who switched to less frequent dosing. More patients who received less frequent dosing ended their clinical trial with a loss of ≥6 letters than patients who received fixed monthly dosing.

 A comparison of baseline characteristics in early 15-letter responders versus delayed 15-letter responders was conducted to determine if any of these characteristics could be predictors of VA response. Baseline VA was lower in early 15-letter responders than in delayed 15-letter responders in all four trials analyzed; these differences were statistically significant in ANCHOR and MARINA. In subgroup analyses of ANCHOR and MARINA, baseline VA was the most important predictor of VA outcomes at later time points [12, 13]. Because no other baseline characteristics were significantly different between early and delayed 15-letter responders, and the difference in baseline VA was statistically significant in only two of the four clinical trials analyzed, baseline characteristics may not provide a good prediction of early versus delayed visual response. As demonstrated in the results of this analysis, month 3 VA may provide some indication of VA at months 6, 12, and 24, particularly if the patient is maintained on fixed monthly dosing. Since not all patients have achieved optimal gains at month 3, use of month 3 VA as a predictor may result in the underestimation of some patients' VA outcomes at later times. 

Although these cross-trial comparisons suggest a difference in VA outcomes between dosing regimens, study populations were different with distinct eligibility criteria and type of lesions eligible for each clinical trial. In addition, some patients were allowed to roll over from quarterly to monthly ranibizumab 0.5 mg during the second treatment year (as early as month 19) in PIER. Accordingly, caution should be taken in comparing results between these clinical trials.

 In conclusion, the results of the current analysis indicate that fixed monthly dosing may produce greater improvement in visual outcomes than when dosing is decreased to less frequent administration following three monthly loading doses. Although other dosing strategies may give similar results as monthly dosing, our clinical trial results suggest that monthly dosing may result in more patients maintaining initial improvements in VA or possibly lead to a greater proportion of patients who did not have clinically significant improvement in VA at month 3 achieving a 15-letter gain later during treatment. These results also suggest that reducing treatment frequency to less frequent dosing may result in underdosing and, consequently, the possibility that the patient's maximal visual potential may not be realized. The CATT study's ranibizumab as-needed treatment arm may propose a compromise. With regular monitoring, visual outcomes between fixed monthly and as-needed treatment arms were similar. With this evaluation method, dosing of ranibizumab was decreased from 11.7 to 6.9 injections per year [8]; however, regular monthly evaluations appear to be required. Although the current study provides guidance for dosing over the period studied, the duration of fixed monthly dosing required beyond 12 or 24 months for optimal visual outcomes is not currently known. 

## Figures and Tables

**Table 1:** Clinical trial designs.

Fixed monthly dosing regimen	Less than monthly dosing regimen after three monthly loading doses
ANCHOR [1]	PIER [3]
(i) Pivotal, phase III, and double masked	(i) Phase IIIb, double masked
(ii) Predominantly classic CNV	(ii) Minimally classic, predominantly classic, or occult CNV, with or without a classic CNV component
(iii) Three arms: PDT, ranibizumab 0.3 mg, and ranibizumab 0.5 mg	(iii) Three arms: sham injection controlled, ranibizumab 0.3 mg, and ranibizumab 0.5 mg
(iv) Option of crossover from PDT cohort to ranibizumab 0.3 mg in second treatment year	(iv) Option of crossover from sham cohort to ranibizumab 0.5 mg followed by option for all cohorts to roll over from quarterly to monthly ranibizumab 0.5 mg dosing in second treatment year
(v) Dosing: monthly for 2 years	(v) Dosing: three monthly loading doses + quarterly maintenance doses for 2 years

MARINA [2]	SAILOR cohort 1 treatment-naïve^a^ [4]
(i) Pivotal, phase III, double masked	(i) Phase IIIb, single masked
(ii) Minimally classic or occult without classic CNV	(ii) All CNV subtypes, with evidence of recent disease progression
(iii) Three arms: sham injection controlled, ranibizumab 0.3 mg, and ranibizumab 0.5 mg	(iii) Two arms: ranibizumab 0.3 mg, ranibizumab 0.5 mg
(iv) Option of crossover from sham cohort to ranibizumab 0.5 mg late in second treatment year	(iv) Dosing: three monthly loading doses + retreatment as needed (quarterly scheduled monitoring visits) based on VA and/or OCT, for 1 year
(v) Dosing: monthly for 2 years	

CNV: choroidal neovascularization; OCT: optical coherence tomography; PDT: photodynamic therapy; VA: visual acuity.

^a^For SAILOR, only cohort 1 treatment-naïve patients were included in this analysis. This ensured that the patient population was most similar to the ANCHOR, MARINA, and PIER trials, and that all clinical trials included in this analysis began with three monthly loading doses as part of their dosing schedules.

**Table 2:** Baseline characteristics of early versus delayed 15-letter responders.

	Fixed monthly dosing				Less frequent dosing			
	ANCHOR		MARINA		PIER		SAILOR cohort 1 treatment-naïve at Baseline	
	Early 15-letter	Delayed 15-letter	Early 15-letter	Delayed 15-letter	Early 15-letter	Delayed 15-letter	Early 15-letter	Delayed 15-letter
	responders	responders	responders	responders	responders	responders	responders	responders
	(*n* = 82)	(*n* = 41)	(*n* = 87)	(*n* = 77)	(*n* = 18)	(*n* = 6)	(*n *= 187)	(*n *= 57)
Demographics								
Gender, % female	46	54	63	66	39	50	56	51
Age, mean years	76	75	77	76	78	80	78	79
VA, mean letters	43*	51	47*	53	46	49	49	52
Prior therapy for AMD,^a^ %	17	17	9	5	22	0	0	0
Total area of, mean DA								
Lesion	1.7	1.6	4.0	4.2	4.1	1.7	NA	NA
CNV	1.3	1.2	3.8	4.1	3.4	1.5	NA	NA
Classic CNV	1.1	1.1	0.2	0.2	0.7	0.1	NA	NA
Classic CNV, % of lesion area	65	69	5	4	32	14	NA	NA
Leakage + RPE staining	2.8	2.5	3.1	3.4	3.8	2.2	NA	NA

*P < 0.05 comparing early 15-letter responders versus delayed 15-letter responders.

AMD: age-related macular degeneration; CNV: choroidal neovascularization; DA: optic disk areas; NA: not available; RPE: retinal pigment epithelium; VA: visual acuity.

^a^Prior therapies for AMD included extrafoveal photodynamic therapy, laser photocoagulation (juxtafoveal or extrafoveal photocoagulation was allowed 1 month or more preceding day 0 in ANCHOR, MARINA, and PIER), and injections of triamcinolone acetonide and alteplase injection (intravitreal) to displace blood. Note: some study-eye prior therapies were excluded in the ANCHOR, MARINA, PIER, and SAILOR clinical trial protocols.
